# Acid-Base Chemistry of White Wine: Analytical Characterisation and Chemical Modelling

**DOI:** 10.1100/2012/249041

**Published:** 2012-04-01

**Authors:** Enrico Prenesti, Silvia Berto, Simona Toso, Pier Giuseppe Daniele

**Affiliations:** ^1^Dipartimento di Chimica Analitica dell'Università degli Studi di Torino, Via Pietro Giuria 5, 10125 Torino, Italy; ^2^BP Italia S.p.A. Divisione Castrol Industrial, Environment Park, Via Livorno 60, 10144 Torino, Italy

## Abstract

A chemical model of the acid-base properties is optimized for each white wine under study, together with the calculation of their ionic strength, taking into account the contributions of all significant ionic species (strong electrolytes and weak one sensitive to the chemical equilibria). Coupling the HPLC-IEC and HPLC-RP methods, we are able to quantify up to 12 carboxylic acids, the most relevant substances responsible of the acid-base equilibria of wine. The analytical concentration of carboxylic acids and of other acid-base active substances was used as input, with the total acidity, for the chemical modelling step of the study based on the contemporary treatment of overlapped protonation equilibria. New protonation constants were refined (L-lactic and succinic acids) with respect to our previous investigation on red wines. Attention was paid for mixed solvent (ethanol-water mixture), ionic strength, and temperature to ensure a thermodynamic level to the study. Validation of the chemical model optimized is achieved by way of conductometric measurements and using a synthetic “wine” especially adapted for testing.

## 1. Introduction

Nowadays the wine has achieved considerable importance in the global economy. The chemical characterization aimed to deliver an excellent product to the consumer assumes a growing importance in the world of oenological analysis. Particularly, the acidity of wine is a characteristic of central importance. Acid-base chemistry of wines is mainly based on the protonation/deprotonation *status* of the carboxylic acids. Carboxylic acids belong to a class of natural compounds widespread distributed in fruits and vegetables. Moreover, they are extensively used as food acidulants in the manufacturing of beverages, fruit- and vegetable-based drinks or juices, and as antioxidants, acidifiers and drug adsorption modifiers in pharmaceutical industries. Physical and chemical analyses of wines have become one of the most important aspects of the modern quality control in the food field. Particularly, in enology the nature and concentration of the carboxylic acids are very important for (i) acid-base and redox properties, (ii) biological and colour stability, (iii) organoleptic characteristics, and (iv) monitoring of fraudulent practices and/or alterations [1].

 In our previous paper on red wines [2] we needed to quantify all acid-base active substances in order to investigate the acid-base chemistry of red wine at a speciation level by way of a careful equilibrium analysis. A chemical model was proposed, based on the simulation of the alkalimetric titration curve of each wine. The simulated titration curve was obtained by way of a multicomponent equilibrium calculation made thanks to suitable values of protonation constants of significant pH-determining substances found in wines. An HPLC-RP separation of carboxylic acids and proline was executed, improving the method of Tusseau and Benoit [3]. Unfortunately, a coelution of acetic and 2-ketoglutaric acids was observed under the adopted experimental conditions. To ensure the separation of these two acids, we have now used an ion-exclusion chromatographic (HPLC/IEC) separation [4, 5] with photometric detection. We propose improvements in both analytical and equilibrium aspects of our study. Coupling the current HPLC-IEC method with previous HPLC-RP one, we are now able to quantify up to 12 carboxylic acids. Further protonation constants, with respect to those previously determined [2, 6], were now refined (at 25°C in mixed ethanol-water solvent in KCl 0.05 or 0.1 M)—L-lactic and succinic acids—to strengthen the thermodynamic approach to the acid-base chemistry of wines. The analytical concentration of carboxylic acids, and other inorganic and organic acid-base active substances, was used as input, with the total acidity, for the chemical modelling step of the study, assisted by a specific software based on the contemporary treatment of overlapped protonation equilibria. Attention was paid for mixed solvent (ethanol-water mixture), ionic strength, and temperature, to ensure a thermodynamic level to the study.

 Six white wines were included in this investigation: (i) four white wines are produced in Piedmont (North-West Italy), named Erbaluce and Cortese; two vintages were considered for these wines, that is, Erbaluce 2007 and 2008 (henceforth E07 and E08) and Cortese 2007 and 2008 (henceforth C07 and C08); (ii) one Californian wine: Chardonnay 2009, a white wine produced in Sonoma Valley (San Francisco) (henceforth Ch09); (iii) one French white wine, produced in Alsace, namely, Riesling 2007 (henceforth R07).

 A chemical model is optimized for each white wine together with the calculation of its ionic strength taking into account the contributions of all significant ionic species (strong electrolytes and weak one sensitive to the chemical equilibria). Validation of the chemical model is achieved by way of conductometric measurements and using a synthetic “wine” especially adapted for testing.

## 2. Experimental

### 2.1. Sample Storage

All wines were stored at room temperature in the dark and were subdivided into small glass bottles (250 mL capacity) to avoid air contact and other contamination.

### 2.2. Reagents and Solutions

Standard solutions of carboxylic acids were prepared from analytical-reagent grade chemicals; standardization process was performed to achieve high-quality, by titrating each stock solution with standard carbonate-free KOH.

 Carboxylic acids: 2-ketoglutaric, shikimic, L-lactic, L-citramalic, and gallic acids were from Sigma; L-tartaric and succinic acids were from Carlo Erba; DL-malic, acetic, and citric acids were from Merck; pyruvic and fumaric acids were from Fluka. Amino acid: L-proline was from Sigma. Salts: KH_2_PO_4_ was from Sigma, (NH_4_)_2_SO_4_ was from Carlo Erba. Solvents: acetone was from Labochem, ethanol (96% w/v) was from Merck. Mineral acids: phosphoric acid (85% w/v) was from Carlo Erba, sulfuric acid (95–97% w/v) was from Fluka. Standard solutions of KOH were prepared by diluting concentrated Merck ampoules and were standardised against potassium hydrogen phthalate (Fluka, puriss.). Amberlite (IR-124), a cation exchange resin, was from Carlo Erba.

Grade A glassware and deionised and twice distilled water were used for all solutions.

### 2.3. Chromatographic Apparatus for Carboxylic Acids Analysis

Chromatographic analyses for carboxylic acids were carried out with an Agilent 1100 chromatograph equipped with an Agilent 1100 pump. The injector was a rheodyne valve with a 20 *μ*L sampling loop, and the detector was an Agilent 1100 UV-vis photometer.

 As to HPLC-RP analyses, the chromatographic separations were performed on a Merck Superspher 100 RP-18 end-capped (250 × 4 mm I.D.) spherical phase column with a particle size of 4 *μ*m.

 The HPLC-IEC analyses were carried out using a Supelcogel C-610H column (300 × 7.8 mm I.D.), polystyrenedivinylbenzene-based strong acid cation exchange resin in the H^+^-form (9 *μ*m particle size).

### 2.4. Chromatographic Conditions for Carboxylic Acids Analysis

As for HPLC-RP separation, the mobile phase was composed of 70 g/L (0.52 M) potassium dihydrogen phosphate and 14 g/L (0.10 M) ammonium sulphate adjusted to pH 2.1 with phosphoric acid, in order to have the highest protonation degree of the acids under examination, according to the method of Tusseau and Benoit [3]. The flow rate was 0.7 mL/min at room temperature. The separation was carried out under isocratic conditions, and the detection was effected by measurement of the UV absorption at 210 nm. See further details in [2].

 For HPLC-IEC separation, the mobile phase was composed of 10% acetone and 0.5 mM sulphuric acid. The flow rate was 0.4 mL/min. Column and precolumn were thermostated at 45°C. The separation was carried out under isocratic conditions, and the detection was effected by measurement of the UV absorption at 210 nm.

 Each substance determined via HPLC was identified by its retention time in comparison with the response of standard solution of pure compounds. Standard addition of some substance to the wine was performed in order to verify the attribution of the peaks.

### 2.5. Preliminary Treatments of Wine Samples for Carboxylic Acids Analysis

Both for HPLC-RP and HPLC-IEC the samples were treated by means of Chromabond C18 (500 mg) MACHEREY-NAGEL cartridges, which allow to purify the matrix of those molecular compounds (polyphenols and saccharides, as an example) which can interfere in the chromatographic measurements. This treatment does not alter the carboxylic acid composition of the samples, as verified by means of a check on synthetic mixtures. Each cartridge was conditioned with little volume (few mL) of water and then with little volume (few mL) of ethanol, before sample purification procedure. As for ion-exclusion chromatography, each sample was also purified from metal ions in order to prevent the saturation of the cation exchange resin surface of the column used for the separation. 1 g of a cation exchange resin (Amberlite, IR-124) was added to 100 mL of each wine and stirred for twenty minutes by means of a magnetic stirrer. Finally, each sample was filtered through a 0.45 *μ*m Millipore filter and diluted 1/10 (v/v) with twice distilled water before the chromatographic injection.

### 2.6. Ion Chromatography for Inorganic Anions Analysis

As for inorganic anions, analysis was carried with a Metrohm 690 Ion Chromatograph equipped with a Bischoff HPLC pump. The chromatographic separations were performed on a PRPTM-X100 column (polystyrenedivinylbenzene-trimethylammonium exchanger spherical phase column). The separation was performed at pH ≈ 8. The mobile phase was composed of 4 mM *p*-hydroxybenzoic acid and 1% methanol adjusted to pH 8.5 with NaOH (in order to have maximal deprotonation of the analytes). The flow rate was 1.5 mL/min at room temperature.

### 2.7. ICP/AES

A model Liberty 2 ICP/AES was used to determine metal ions in wine. Potassium, sodium, calcium, magnesium, and iron were determined after suitable dilution of each sample, depending upon the nature of the analyte. The quantification was based on external standards.

### 2.8. Potentiometric Apparatus

Potentiometric measurements were performed at *T* = 25 ± 0.1°C and ionic strength *I* = 0.05 or 0.1 M (KCl) with C_2_H_5_OH (EtOH) at 12% level with a model 713 Metrohm potentiometer equipped with a combined glass electrode. The titrant was dispensed with a model 765 Dosimat burette by Metrohm. The electrode couple was calibrated in −log[H^+^] units (pH) employing alkalimetric titrations of hydrochloric acid with standard, carbonate-free, potassium hydroxide. Ionic strength, ionic medium, and ethanol percentage of the calibrating solutions were the same as the solutions being examined. The alkalimetric titrations were carried out in a stream of purified nitrogen gently bubbled in the titration cell. Temperature control was achieved by means of a circulation of water, in the outer chamber of the titration cell, from a model D1-G Haake thermocryostat. Each titration was at least repeated three time.

### 2.9. Preliminary Treatments for Titrations

Before the acid-base titration each wine was filtered through a 0.45 *μ*m Millipore filter; CO_2_ was then removed by means of strong stirring under vacuum (few minutes), according to the indications of official methods [8].

### 2.10. Equilibrium Calculations

The nonlinear least squares computer program ESAB2M was used to evaluate the purity of the reagents (starting from acid-base titration data) and to refine all the parameters related to the calibration of the electrode system [9].

The protonation constant values were expressed by the general formula: *β*_*pq*_ = [*L*_*p*_ *H*_*q*_]/[*L*]^*p*^[*H*]^*q*^ and refined (as log⁡*β* ^*H*^) by means of the BSTAC program, which minimises the error squares sum on electromotive force values and is able to take into account (if desired) eventual variations of ionic strength among and/or during titrations [10].

Distribution diagrams and simulated titration curves were obtained using the computer program ES4ECI [10].

The slight contraction in volume around 0.3–0.4% [2], corresponding to the ethanol-water mixture formation, was considered in all measurements and calculations.

### 2.11. Conductometric Apparatus

A model 160 Amel conducimeter equipped with a model 196 Amel electrode, was used as indicating device during alkalimetric titration of wines (*T* = 25°C).

### 2.12. Alcohol Content

The alcohol (ethanol) contained in each wine (symbol “% vol.”) was determined, according to the official method [8], by means of distillation and further density measurement (20°C).

## 3. Results and Discussion

### 3.1. Analytical Determinations

#### 3.1.1. Quantification of Organic Acids

**Figure 1:**
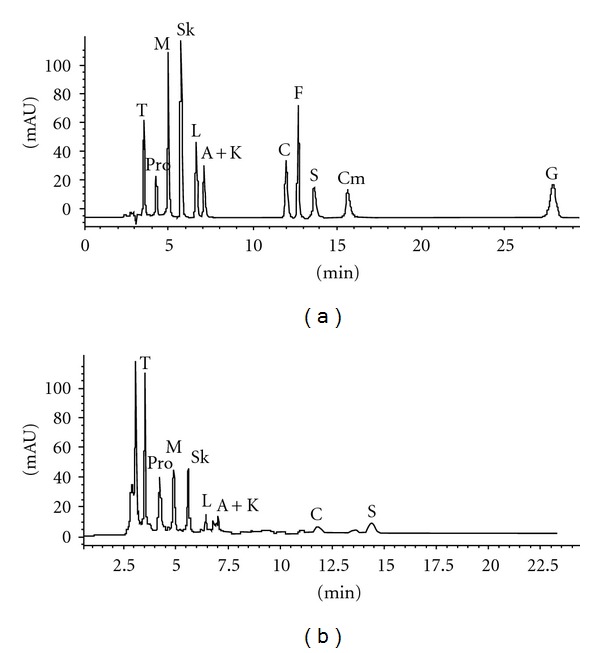
HPLC-RP separation: (a) chromatogram of a standard mixture of carboxylic acids. Concentrations in mg/L are T 100, Pro 250, M 250, Sk 5, L 250, A 150, K 100, C 250, F 3, S 550, Cm 300, G 2.5. (b) Chromatogram of Ch09 (dilution 1/10 v/v). Run stopped at 22.5 min.

First, to quantify carboxylic acids in wine, we chose to adopt a method without derivatization based on a classical RP separation (as in our previous paper [2]). The conditions adopted allowed us the separation of the following acids (in parentheses, the retention time is indicated): tartaric (3.6 min), malic (5.0 min), shikimic (5.8 min), lactic (6.7 min), citric (11.9 min), fumaric (12.8 min), succinic (13.7 min), citramalic (15.7 min), and gallic (27.9 min), together with the simultaneous determination of the proline (4.3 min), the most abundant amino acid present in wine [1]. In Figure 1(a) a standard run is shown. Calibration graphs (peak height or area versus substances concentration) were built in order to characterize the acidic profile of wines under investigation, starting by a chromatographic run of a stock solution and its further dilutions. In Figure 1(b) the chromatographic separation on Ch09 is given as an example. Unfortunately, a coelution of acetic and 2-ketoglutaric acids is observed under the adopted experimental conditions at a *t*_*R*_ of 7.2 minutes. Plot of the integrated peak area and/or height against concentration (mg/L) of each molecule was always linear (the correlation coefficient *R* ranged between 0.9997 and 0.9999) in the concentration ranges investigated.

 With HPLC-IEC the conditions adopted allowed us the separation of (in parentheses, the retention time is indicated) 2-ketoglutaric (10.8 min), pyruvic (11.8 min), and acetic (23.2 min). Calibration graphs were obtained by plotting peak height or area against analytes concentration and excellent linearity was found for each standard molecule, being *R* in the range 0.9992 ≤ *R* ≤ 1. The repeatability of peak heights or areas, obtained by repeated (at least three) injections (HPLC-IEC) of the same concentration of standard, is in the range 0.6–4% (relative standard deviation) for all the carboxylic acids studied. Intraday and infraday repeatability was of very similar extent.

 In Table 1, the concentration (mg/L) of carboxylic acids utilized for the optimization of the chemical model is collected for each wine under investigation. Comparing the results with those obtained on red wines analysed in the previous paper [2], in white wines a higher concentration of citric acid was measured while citramalic and gallic acids are found only in the red wines previously analysed.

#### 3.1.2. Quantification of Inorganic Anions and Cations

Results for inorganic anions and cations are collected in Tables 2 and 3 respectively.

The inorganic anions were determined with HPLC-IC, and the conditions adopted allowed us the separation of (in parentheses, the retention time is indicated) phosphate (7.1 min), nitrate (6.3 min), sulphate (3.9 min), and chloride (3.2 min). Calibration graphs were obtained by plotting peak area against analytes concentration, and excellent linearity was found for each standard molecule, being *R* in the range 0.9993 ≤ *R* ≤ 0.9998. The results reported in Table 2 show that sulphates are predominant, while nitrates were found in low concentration only in Ch09 and chlorides resulted under the detection limit.

As to cations, the results reported in Table 3, obtained by ICP/AES, show that potassium is the predominant one in all wines.

### 3.2. Equilibrium Determinations

#### 3.2.1. Protonation Constants in Mixed Solvent

In our previous paper [2] three carboxylic acids—acetic, L-tartaric, and citric acid—chosen as model substances, were titrated in order to refine the overall protonation constant values (log⁡*β* ^*H*^) for mono-, di-, and triprotic acids in ethanol-water media. We assumed that the differences experimentally observed (Δlog⁡*β* ^*H*^) for each of our three model molecules in water and ethanol-water media are the same under the same conditions of electrical charges involved in any single-step protonation reaction. The terms Δlog⁡*β* ^*H*^ were applied to calculate the values of log⁡*β* ^*H*^ for the substances not titrated, starting by the values in aqueous solution. Now, we added L-lactic and succinic acids to improve the model by verifying previous assumptions with common substances present in wine but lightly different from those previously considered. Conditions: KCl at two ionic strength values, *I* = 0.05 and *I* = 0.1 M, was used as background electrolyte in 12% ethanol-water mixture. Since K^+^ ion is the most representative metal ion in wines [1], we believe the choice of KCl as background salt to obtain a suitable set of protonation constant values to model the acid-base chemistry of wines is correct. These experimental values of log⁡*β* ^*H*^ in 12% ethanol-water mixture were reported in Table 4 and were compared with those obtained with the calculation previous described [2]. The comparison is satisfactory. The log⁡*β* ^*H*^ in aqueous medium were also collected in Table 4. The values at *I* = 0.1 M are from [7], whereas the values at *I* = 0.05 M are calculated by way of a Debye-Hückel-type equation as reported in [2]. Any chemical model developed in this paper is based on the set of protonation constants presented in [2] with the integration currently obtained for L-lactic and succinic acids (Table 4).

#### 3.2.2. Alkalimetric Titration of Wines

Table 5 reports pH, alcoholic grade and total acidity for each wine. CO_2_ was preliminarily removed by means of brief stirring under vacuum. The pH was measured on undiluted wines, at *T* = 25°C, and expressed as −log[H^+^]. Wine was diluted 1 : 20 (v/v) for total acidity measurement. During the alkalimetric titration of each wine, we found via potentiometric detection (combined glass electrode) the first inflection point at about pH 7.5, as expected. The strong base used up to this flex allows the calculation of the total acidity parameter *C*_*H*_ (also expressed as g/L of tartaric acid), fundamental in the chemical modelling step of this work.

 Conductometric data were also recorded, during the alkalimetric titrations of each wine. As in potentiometry, the derivative graph can be used to estimate the end point in the conductivity versus titrant volume curve. The estimations of the *C*_*H*_ obtained by potentiometry and conductometry are in excellent agreement (Table 5).

### 3.3. Chemical Modelling

#### 3.3.1. Building of the Chemical Model

For each wine a chemical model can be built taking into account the analytical concentrations of any acid-base active substance analysed and the refined values of the protonation constants in the suitable chemical medium. As to current wines, a greater amount of SO_2_ is found with respect to red wines previously investigated [2]. Nevertheless, the most of SO_2_ is in the combined form (as Bertagnini adduct), and it does not affect the acid-base equilibria for pH < pH_endpoint_. Moreover, the contribution of the free SO_2_ (measured by titration with I_2_, according to the official methods [8]) resulted negligible considering that the p*K*_a2_ = 6.85 of H_2_SO_3_ (25°C, *I* = 0.1 M in water) hinders its fully deprotonation at the experimental pH value of the endpoint of the titration curve of wine. Similar behaviour is evidenced for phosphoric acid (p*K*_a2_ = 6.75, 25°C, *I* = 0.1 M in water).

 13 acid-base active substances (in the field of pH < pH_end point_) were considered as reactants during the input building for the computer-assisted simulation of the alkalimetric titration of each wine, 5 metal ions (and nitrate ions for Ch09) were considered as background electrolytes (contributing to the ionic strength of each fluid), and 20 protonation equilibria were treated at the same time. We considered the hydrolysis of iron(II) (wine is under reducing conditions), but the influence on pH calculation is not appreciable. The model investigation was stopped at pH ≈ 6.5.

 The pH value of each wine was then calculated (pH_calc_) as a result of all the multiple chemical protonation equilibria set.  Table 6 shows the pH_calc_ values for each wine obtained by various simulations. Four chemical models were tested considering the following: (i) Model 1: the values of each log⁡*β* ^*H*^ in water at *I* = 0.05 M (KCl), (ii) Model 2: the values of each log⁡*β* ^*H*^ in water at *I* = 0.1 M (KCl), (iii) Model 3: the values of each log⁡*β* ^*H*^ at *I* = 0.05 M (KCl) in 12% ethanol, and (iv) Model 4: the values of each log⁡*β* ^*H*^ at *I* = 0.1 M (KCl) in 12% ethanol.

 The best simulation of the acid-base chemistry of each wine is obtained by the set of thermodynamic data corresponding to Model 3. The outputs recorded in Table 6 clearly show the importance of the ethanol, while the role of the concentration of the background salt, in the range investigated, is of minor relevance.

**Figure 2:**
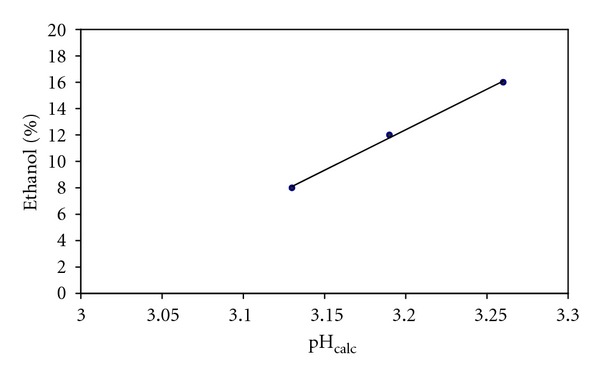
Linear fitting applied in order to estimate the correct value of pH at the real value of % ethanol for Ch09. The equation model resulted *y* = 61.417*x* − 184.13 with *R* = 0.999.

 For the Ch09, which has an alcohol content significantly exceeding 12% vol., we took into account the influence of the amount of ethanol by applying the corrective procedure previously described [2]. The method allows to avoid the use of the specific set of log⁡*β* ^*H*^ values at the current alcoholic grade. We used the avalilable set of log⁡*β* ^*H*^ at 8, 12, and 16% of ethanol (*I* = 0.05 M, KCl) [2] to calculate three pH values for each wine. A linear fitting was applied to the points of the diagram % ethanol versus pH (see Figure 2), and the corrected value of pH was then interpolated. The correction for Ch09 provided pH = 3.21 starting by pH_model3_ = 3.19 (pH_exp_ = 3.21).

 We can estimate the accuracy by way of the difference between measured and calculated pH of each wine: *|*pH_exp_ − pH_calc_ *|*. The average value of six white wines is 0.05, a result that can be considered satisfactory, particularly taking into account the intrinsic uncertainty of the pH reading (±0.02 [11]) and the wide variety and amount of data—of both analytical and thermodynamic nature—used as input in the simulation.

#### 3.3.2. Validation of the Chemical Model

The output based on the chemistry simulated by Model 3 also contains the ionic strength (molar scale) calculated for each wine. As shown in Table 7, the mean value of the ionic strength is around 0.05 M (lightly higher for E07 and E08, near 0.07 M), as that estimated for the red wines previously studied [2]. The trend of the ionic strength with varying the pH in the range 3–6 follows that of the conductivity, indicating the consistency of our assumptions and the applicability of the chemical model optimized. Conductometric and pH-metric outputs show fruitful convergence—experimentally proved by the measurement of the total acidity (Table 5)—and comparable sensitivity with respect to our purposes of overall reliability and accuracy.

**Figure 3:**
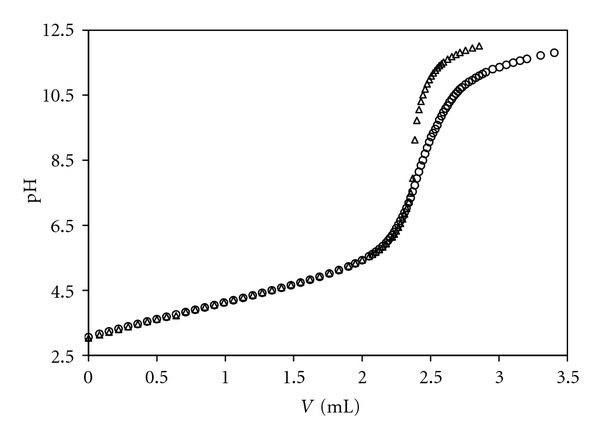
Alkalimetric titration curve (*v* = 25 mL) for natural (○) and synthetic (*▵*) E08 at *T* = 25°C. Titrant: standardized KOH 1.00 M. Natural E08: *C*_*H*_ = 96.00 mM, pH = 3.05. Synthetic E08: *C*_*H*_ = 95.76 mM, pH = 3.03.

 To reach an experimental confirmation of our model, a synthetic solution reproducing the composition of a wine, with respect to the acid-base reactivity (until pH < 7), was prepared. A mixture of carboxylic acids, proline, inorganic anions and cations, ethanol, and water was achieved (using standardized solutions of each significant reactant) for E08, thus faithfully reproducing the content of bases (organic and inorganic ones) and the values of *C*_*H*_, ionic strength, percentage of ethanol. The synthetic mixture was alkalimetrically titrated, as the wines.  Figure 3 shows the overlap of the titration curves for natural and synthetic E08 showing an excellent agreement up to pH 7.

## 4. Conclusions

The use of multitechnique analytical and equilibrium measurements combined with a chemical modelling step allowed us to develop a thermodynamic approach to the acid-base chemistry of white wine. Since the chemical modelling is mainly based on the chemistry of carboxylic acids, particular attention was paid to quantify these substances. With respect to the past, we added HPLC-IEC to HPLC-RP to improve the characterization of the carboxylic fraction. We can observe that HPLC-RP and HPLC-IEC are complementary techniques: joining the separation ability reached with these two methods we are now able to quantify up to 12 carboxylic acids in wine. Conductometric results, together with the information obtained by the synthetic “wines”, contributes to validate our approach and assumptions. Switching from the analytical to the equilibrium composition allows the prediction of the effect on wines of oenological treatments (as addition of acid-base active substances) or natural transformations (as precipitation or redox reactions). Many characteristics of wine, specially those related to the ageing process, are always dependent from acid-base conditions. Moreover, this investigation at molecular and thermodynamic level contributes to the basic knowledge, and the science presented in this paper can also be used as an input for model speciation building of other natural fluids.

## Figures and Tables

**Table 1:** Analytical concentrations (mg/L) of carboxylic acids and proline quantified in each wine under investigation by HPLC-RP and HPLC-IEC. The relative standard deviation, evaluated on three replicates on each sample, ranges between 0.6 and 10%.

Substances	Identification Symbol	Wine					
		E07	E08	C07	C08	Ch09	R07
Acetic acid	A	395.3^(a)^	349.1^(a)^	710.1^(a)^	528.1^(a)^	366.5^(a)^	240.3^(a)^
Citramalic acid	Cm	<d.l.^(b)^	<d.l.	<d.l.	<d.l.	<d.l.	<d.l.
Citric acid	C	718.5	257.4	499.5	426.5	480.3	326.6
Fumaric acid	F	<d.l.	<d.l.	<d.l.	<d.l.	<d.l.	<d.l.
Gallic acid	G	<d.l.	<d.l.	<d.l.	<d.l.	<d.l.	<d.l.
2-Ketoglutaric acid	K	58.4^(a)^	190.0^(a)^	24.8^(a)^	33.6^(a)^	26.3^(a)^	23.4^(a)^
Lactic acid	L	614.5	653.8	1116.6	1000.4	758.5	374.4
Malic acid	M	2419.0	3038.5	449.2	682.5	1702.9	2373.4
Proline	Pro	300.4	308.5	545.6	527.2	4109.1	1922.2
Pyruvic acid	Py	201.5^(a)^	236.7^(a)^	214.7^(a)^	196.2^(a)^	44.9^(a)^	13.2^(a)^
Shikimic acid	Sk	34.8	33.1	20.9	13.9	34.8	52.3
Succinic acid	S	916.4	866.8	395.6	560.9	259.8	236.2
Tartaric acid	T	1805.6	2053.2	1900.1	1678.0	2926.8	4247.5

^(a)^Data obtained from HPLC-IEC technique.

^(b)^d.l. = detection limit.

**Table 2:** Concentration (mM) of inorganic anions in each wine. The uncertainty (three replicates) ranges between 2 and 10% (±*s*).

Inorganic anion	Wine					
	C07	C08	E07	E08	Ch09	R07
Phosphate	3.77	3.95	4.18	4.47	5.22	3.28
Nitrate	<d.l.^(a)^	<d.l.	<d.l.	<d.l.	0.26	<d.l.
Sulphate	14.04	12.48	17.49	17.88	4.83	3.46
Chloride	<d.l.	<d.l.	<d.l.	<d.l.	<d.l.	<d.l.

^(a)^d.l. = detection limit.

**Table 3:** Concentration (mM) of the metal ions in each wine (ICP/AES). The uncertainty (three replicates) ranges between 2 and 8% (±*s*).

Metal ion	Wine					
	C07	C08	E07	E08	Ch09	R07
Ca	2.2	2.2	2.5	2.5	1.64	2.70
Fe	0.04	0.03	0.09	0.05	0.04	0.04
K	13.2	11.5	22.8	16.9	12.93	17.96
Mg	3.1	2.9	3.4	3.6	4.27	3.05
Na	1.3	1.3	0.7	0.5	1.33	0.75

**Table 4:** Overall protonation constant values, as log *β*_*i*_^*H*^, at two ionic strength values (0.05 and 0.1 M), 0% and 12% of ethanol, K^+^Cl^−^ as background salt, *T* = 25°C. The uncertainty is reported in parentheses as standard deviation in the last significant digit.

Substance	*i*	log *β*_*i*_^*H*^ (KCl 0.05 M)			log *β*_*i*_^*H*^ (KCl 0.1 M)		
		EtOH 0%	EtOH 12%		EtOH 0%	EtOH 12%	
L-Lactate	1	3.70^(a)^	3.870 (3)	3.81^(b)^	3.66^(c)^	3.830 (3)	3.77^(b)^
Succinate	1	5.319^(a)^	5.49 (2)	5.467^(b)^	5.24^(c)^	5.39 (2)	5.38^(b)^
	2	9.347^(a)^	9.68 (5)	9.651^(b)^	9.23^(c)^	9.59 (3)	9.53^(b)^

^(a)^Values at *I* = 0.05 M are calculated by way of a Debye-Hückel-type equation from literature data [2].

^(b)^Data from [2].

^(c)^Data from [7].

**Table 5:** Alcohol content (% vol.) and acid-base results of each wine.

Wine	% vol.	pH	Total acidity as *C*_*H*_ (mM)		Total acidity
			Potentiometric detection	Conductometric detection	as g/L Tartaric acid
C07	11.7	3.13	66.60	66.00	4.95
C08	11.8	3.12	65.20	65.20	4.89
E07	12.3	3.24	92.20	92.20	6.92
E08	12.3	3.05	96.00	96.00	7.20
Ch09	13.3	3.21	78.70	79.06	5.90
R07	12.3	3.16	86.40	86.80	6.48

**Table 6:** Chemical modelling. Equilibrium-based simulation at 25°C to obtain a calculated value of pH (pH_calc_) according to the four chemical models depending upon the ionic strength value and the percentage of ethanol.

Wine	% vol.	pH_exp⁡_^(a)^	pH_calc_			
			Model 1	Model 2	Model 3	Model 4
			EtOH 0%, KCl 0.05 M	EtOH 0%, KCl 0.1 M	EtOH 12%, KCl 0.05 M	EtOH 12%, KCl 0.1 M
C07	11.7	3.13	2.85	2.85	2.99	2.99
C08	11.8	3.12	2.86	2.86	2.99	2.99
E07	12.3	3.24	2.98	2.98	3.13	3.13
E08	12.3	3.05	2.86	2.86	3.00	3.00
Ch09	13.3	3.21	3.06	3.06	3.19/3.21^(b)^	3.20
R07	12.3	3.16	3.07	3.07	3.22	3.22

^(a)^pH_exp_ is the experimental pH measured at *T* = 25°C and expressed as −log[H^+^].

^(b)^pH value corrected for % ethanol.

**Table 7:** Values of ionic strength calculated for each wine as output of Model 3.

Wine	C07	C08	E07	E08	Ch09	R07
*I*_calc_ (M)	0.055	0.051	0.070	0.071	0.042	0.041
